# Effect of liver growth factor on both testicular regeneration and recovery of spermatogenesis in busulfan-treated mice

**DOI:** 10.1186/1477-7827-9-21

**Published:** 2011-02-04

**Authors:** Miriam Pérez-Crespo, Eva Pericuesta, Serafín Pérez-Cerezales, Maria I Arenas, Maria VT Lobo, Juan J Díaz-Gil, Alfonso Gutierrez-Adan

**Affiliations:** 1 Dpto de Reproducción Animal y Conservación de Recursos Zoogenéticos, INIA, Ctra de la Coruña Km 5.9, Madrid 28040, Spain; 2 Dpto de Biología Celular y Genética, Universidad de Alcalá, 28871 Alcalá de Henares, Madrid, Spain; 3 Servicio de Bioquímica Experimental, Hospital Universitario Puerta de Hierro Majadahonda, Madrid, Spain

## Abstract

**Background:**

Some adult stem cells persist in adult tissue; however, we do not know how to stimulate stem cells in adults to heal injuries. Liver growth factor (LGF) is a biliprotein with hepatic mitogen activity. Its concentration increases markedly in the presence of any type of liver injury, and it shows in vivo therapeutic biological activity at extrahepatic sites.

**Methods:**

We have analyzed the effect of LGF on the replenishment of germinal cells in the testes of mice injected with busulfan, a common cancer drug that also specifically affects germ line stem cells and spermatogonia. We determined the testicular and epididymal weight, spermatozoal concentration in the epididymis and sperm motility, and performed a histological analysis.

**Results:**

Intraperitoneal administration of LGF was able to partially restore spermatogenesis, as well as sperm production and motility, in mice sterilized with busulfan. LGF treatment in busulfan-treated animals that have suffered a disruption of spermatogenesis can accelerate the reactivation of this process in most of the tubules, as shown in the histological analysis.

**Conclusions:**

Our results suggest a potential use of LGF in the mobilization of testicular stem cells and in the restoration of spermatogenesis after busulfan-induced damage to the testicular germinal epithelium.

## Background

Liver growth factor (LGF) is an albumin-bilirubin complex purified from both rat serum and from patients with hepatobiliary disorders [1]. It has been demonstrated that LGF stimulates cell proliferation with no signs of toxicity or tissue degeneration [2]. LGF is used in some experimental models of hypertension, fibrotic lung disease and Parkinson's disease, showing its potential as an antifibrotic, antihypertensive and neuroregenerative agent [3-5]. It has been proposed to be a novel factor useful for neuronal replacement in neurodegenerative diseases [3]. Few experiments have been carried out to study the role of LGF in testicular regeneration [6]. That study, performed in the rat model, concluded that LGF seems to stimulate testicular regeneration after ethane dimethanesulfonate (EDS)-induced Leydig cell depletion. It prevents the germ cell sloughing and Sertoli cell damage and promotes germinal cell growth. Moreover, LGF stimulates the synthesis of vascular endothelial growth factor (VEGF) and its receptors in testis [7]. In the testis, it may be related to spermatogenesis and Leydig cell physiology, since there is no active angiogenesis in the adult male.

In order to carry out the functional assessment of the role of LGF in the renewal activity of male germ line stem cells, cytotoxic testicular damage was induced in mice by intraperitoneal administration of busulfan. Busulfan is an alkylating agent that adversely affects spermatogenesis in mammals, and it is the drug of choice in the treatment of chronic myelogenous or granulocytic leukemia [8] because its cytotoxic activity results in primary destruction of hematopoietic cells. Nevertheless, some secondary malignancies, such as azoospermia, have been reported in treated patients [9], infertility being the major long-term effect of chemotherapy in males. Many authors have used busulfan to deplete stem cells [10-12]. Unlike other chemicals that destroy differentiated spermatogonia, busulfan is a potent agent that preferentially kills spermatogonial stem cells of several species [13]. It has no effect on DNA synthesis; however, it inhibits the next mitosis when it intoxicates the cells in the G1 phase [12]. High-dose administration of busulfan (40 mg/kg) eliminates germ cells, sterilizes males and causes long-term morphological damage to sperm produced by surviving stem spermatogonia [10,14]. However, other authors have shown that after the administration of a single dose of busulfan, partial recovery of spermatogenesis can take place after two spermatic cycles [15].

In this work, we have studied the effects of the administration of LGF on testicular regeneration in mice previously treated with busulfan.

## Methods

### Experimental groups and treatments

Mature male CD1 mice (3-4 months of age) were divided into the following groups: untreated controls (n = 10); busulfan, administered intraperitoneally (i.p.) (40 mg/kg body weight per dose, two doses at a one-week interval; n = 10); LGF alone, at 1.7 μg/mouse, i.p. (animals were injected twice a week for 2 weeks; n = 15); and busulfan+LGF (same doses as previous groups; n = 18). Mice were kept on a 14L:10 D light cycle. All animal experiments were performed in accordance with the Internal Institutional Animal Care and Use Committee of the Instituto Nacional de Investigación y Tecnología Agraria y Alimentaria (Madrid, Spain). Busulfan (Sigma, St. Louis, MO) was first dissolved in dimethyl sulfoxide (Sigma), after which, an equal volume of sterile distilled water was added to provide the final concentration of 40 mg/kg. The four groups of male animals were analyzed in two independent experiments.

### LGF purification

LGF preparations were lyophilized and stored at 4 -C until used, at which time, aliquots were dissolved in saline for i.p. injection. Before using LGF in these experiments, we checked its activity in vivo at several doses, injecting it into normal rats to establish the dose that produced the greatest stimulation of liver DNA synthesis, as determined by incorporation of 3H-thymidine (New England Nuclear, Dreiech, Germany) into DNA [16]. LGF was quantified by high-performance liquid chromatography [17,18].

### LGF administration

Two doses of 1.7 μg/LGF/mouse were injected i.p. each week for two consecutive weeks; the LGF injections were administered 1 week after the first busulfan injection. Animals were sacrificed by cervical dislocation 63 days after the administration of busulfan. Body weight and testicular and epididymal weight were measured for each male, and sperm concentration and motility were evaluated.

### Histological study

Left testes were prepared by making an incision from the proximal to the distal pole, and fragments of parenchyma were fixed in formalin for 24 h. The immersion-fixed testes were sliced transversely into approximately 3-mm thick strips and processed for paraffin embedding. Sections (5-μm thick) across the seminiferous tubules were deparaffinised, hydrated and stained with hematoxylin and eosin for histological examination. The diameters of 30 randomly selected transverse sections of the round-shaped seminiferous tubules were measured for each animal across the minor axis of their cross-sectioned profiles [19].

### Sperm characteristics

Sperm counts were performed using a Burker haemocytometer. Motility was determined by loading a sperm sample onto a prewarmed (37°C) slide and placing it on the heated (37°C) microscope stage [20]. Percentages of motile spermatozoa were assessed by the Integrated Semen Analysis System (ISAS) (Projectes i Serveis R+D S.L., Valencia, Spain).

### Sperm viability

Percentages of live and dead sperm cells were determined using a live-cell nucleic acid stain, SYBR-14, in combination with the conventional dead-cell nucleic acid stain, propidium iodide [21], according to the staining protocol of the live/dead sperm viability kit (Molecular Probes, Eugene, OR). Briefly, 0.8 μl of 20 mM SYBR-14 working solution and 1.2 μl of 2.4 mM propidium iodide working solution were added to 50 μl of the sperm suspension (2-3 × 10^6 ^sperm cells/ml) and incubated at 37°C for 15 min. Then, 20 μl of the sperm suspension were loaded on a glass slide, covered with a cover slip, and immediately observed under a fluorescent microscope equipped with appropriate filters. SYBR-14 stains the nucleus of live sperm green, while dead or membrane-damaged spermatozoa are stained red by the propidium iodide. At least 500 cells were counted per treatment.

### Statistical analysis

Statistical analyses were performed using SigmaStat version 3.1.1 software (Jandel Scientific, San Rafael, CA). Data are given as the mean ± SEM. Comparison of the differences between the means for each treatment was done using ANOVA, followed by the Holm-Sidak method.

## Results

### Testis and epididymidis weight and sperm quality parameters

It has been reported elsewhere that a single dose of busulfan can permanently sterilize mice at nonlethal doses [10]. We have previously demonstrated that CD-1 mice treated with two doses of 40 mg/kg of busulfan remain sterile 70 days after the administration of the drug.

**Figure 1:**
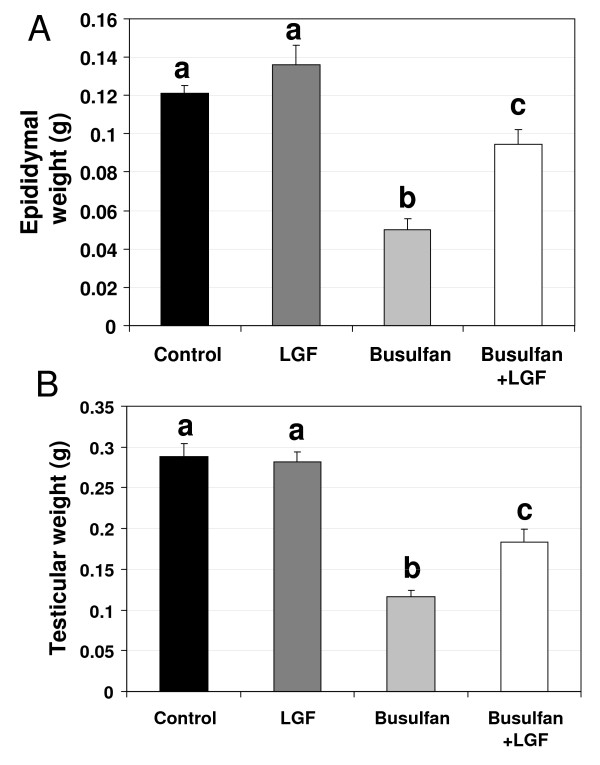
**Differences in testis and epididymis weight observed between experimental groups**. Epididymal (A) and testicular (B) weight in the four groups of animals analyzed (control, LGF treatment, busulfan treatment, busulfan plus LGF treatment). ^a, b, c^Different letters indicate significant differences between groups based on one-way ANOVA (P ≤ 0.01). Error bars, SEM. LGF: liver growth factor.

**Figure 2:**
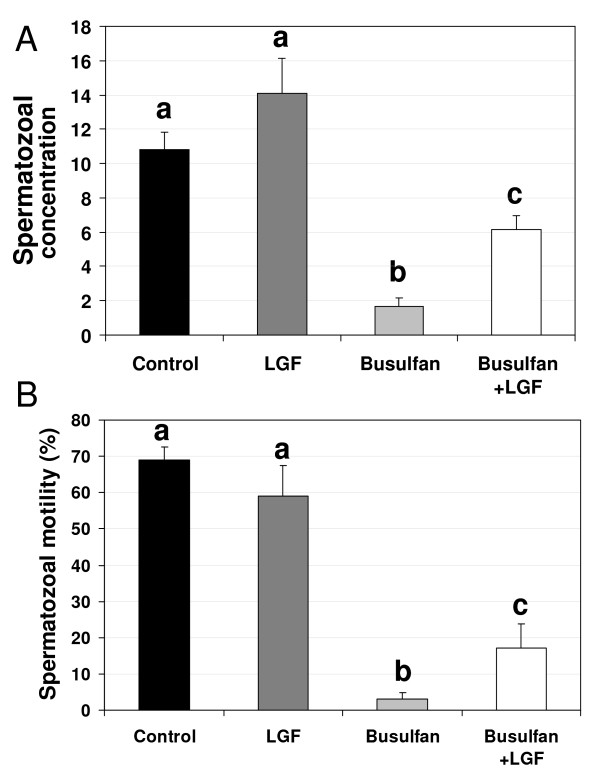
**Differences in sperm motility and concentration observed between experimental groups**. Spermatozoal concentration (×10^6 ^sperm per ml) (A) and motility (B) in sperm samples recovered from cauda epididymidis and vasa deferentia of the four groups of males analyzed (control, LGF treatment, busulfan treatment, busulfan plus LGF treatment). ^a, b, c^Different letters indicate significant differences between groups based on one-way ANOVA (P ≤ 0.01). Error bars, SEM. LGF: liver growth factor.

No significant differences were observed between the LGF and control groups in terms of either testis and epididymis weight (Figure 1) or sperm motility and concentration (Figure 2), indicating that LGF treatment did not induce either an increase in testis and epididymidis weight or an improvement in sperm quality in a normal testis. As observed by other authors [10], mice analyzed 70-63 days after the administration of the first-second doses of 40 mg/kg busulfan showed both a decrease in testis and epididymidis weight and a decrease in sperm concentration and motility when compared with the control and the LGF group (Figures 1 and 2).

When animals treated with busulfan were injected with LGF, there was a decrease in the parameters analyzed compared to the control and LGF groups. However, testis and epididymidis weight and sperm concentration were significantly higher than the values observed in animals treated with busulfan alone. Sperm motility in the animals treated with busulfan and LGF was different from the value observed in mice treated with busulfan alone; this value was also lower than that observed in the control and in the LGF groups (Figure 2).

### Histological study

**Figure 3:**
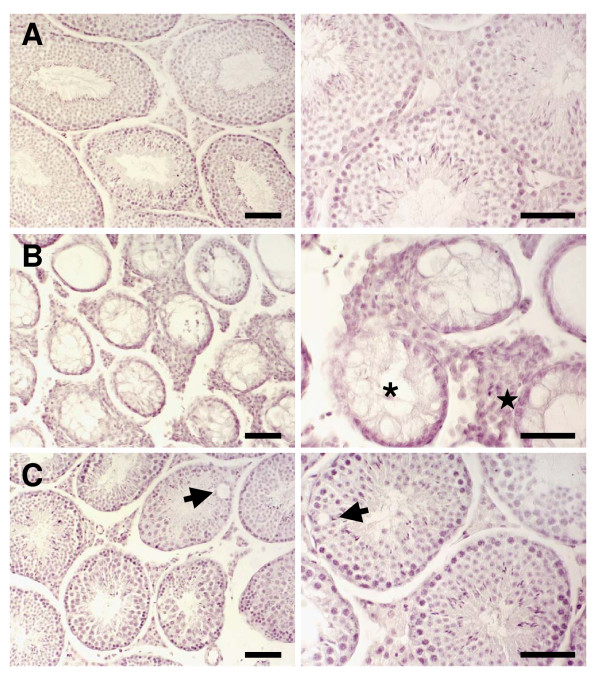
**Histological comparison of seminiferous tubules from testes of experimental groups**. Pictures of the control group (A); 63 days after busulfan treatment (B) with Sertoli cell-only tubules (sterisk) and nodules of hyperplastic Leydig cells (star); and 63 days after the injection of busulfan and liver growth factor (C) with tubules showing vacuolization (arrow). Hematoxylin & eosin. Bar 50 μm.

**Figure 4:**
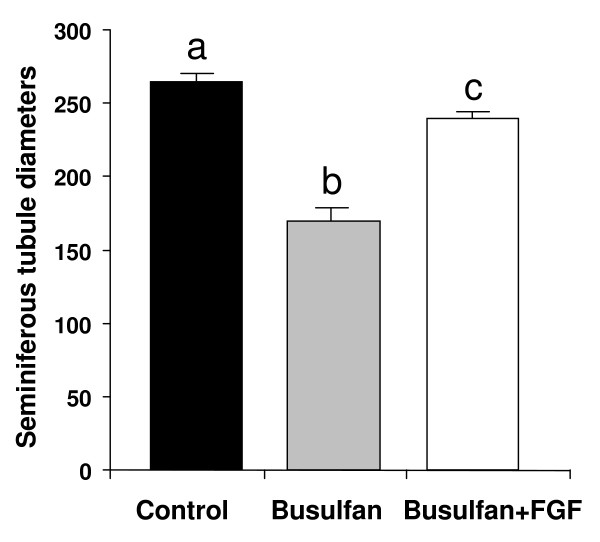
**Seminiferous tubule diameters from the three groups of mice analyzed**. Control, busulfan treatment, busulfan plus LGF treatment. ^a, b, c^Different letters indicate significant differences between groups based on one-way ANOVA (P ≤ 0.01). Error bars, SEM. LGF: liver growth factor.

Histological analysis of testes from animals of the control group is represented in Figure 3A. Histological analysis of testes 63 days after busulfan treatment showed a reduction in the diameter of the seminiferous tubules and several morphological abnormalities such as germ cell sloughing, tubule plugging, vacuolization and Sertoli cell-only tubules (Figure 3 and 4). In the interstitium, areas with nodules of hyperplastic Leydig cells were also observed (Figure 3B). In the group treated with busulfan and LGF, a recovery of germ cells was detected in most of the tubules, although some of them showed disruption of the germinal epithelium (Figure 3C). The hyperplasia of Leydig cells was still observed, but it was not as evident as that observed in the group treated with busulfan alone.

## Discussion

In this study, we show that LGF treatment in control mice has no effect on either testicular and epididymal weight or on sperm quality. However, LGF treatment applied to animals that have been subjected to testicular damage involving the germinal epithelium seems to induce a regeneration of the testis that is reflected in an increase in the weight of testis and epididymis. It also seems that LGF treatment in busulfan-treated animals that have suffered a disruption of spermatogenesis can accelerate the reactivation of this process in most of the tubules, as shown in the histological analysis. This reactivation is also demonstrated by the increase in the sperm concentration value compared to that observed in the animals treated with busulfan alone.

Spermatogenesis is dependent on a population of cells called spermatogonial stem cells. Their number in the testis is very small, and is estimated to be about 2-3 × 10^4 ^in mice [14]. After busulfan treatment, stem cell expansion starts immediately and the doubling time for stem cells is 1-5 weeks [14]. The doses of busulfan used in this work (40 mg/kg) were previously shown to achieve complete depletion of germ cells, but only partial depletion of testicular stem cells to allow spontaneous recovery to occur [15]. We observed the expected responses from the busulfan-treated group. Moreover, LGF affects spermatogenic recovery after injury induced experimentally by busulfan. The remaining spermatogonial stem cells are capable of inducing spermatogenic recovery, and we propose that LGF activates these stem cells to induce a faster recovery of spermatogenesis. As previous authors have reported [22], the histological study showed that busulfan treatment apparently did not affect Sertoli or spermatogonial stem cells, but some morphological abnormalities of the seminiferous tubules were noted. The recovery of the normal histological structure after LGF administration to animals treated previously with busulfan showed not only a reactivation of the spermatogenic process, but also the regeneration of the microenvironment necessary for successful spermatogenesis. It could be also speculated that LGF may have a role in normal development of spermatogenesis, as previously has been reported for the hepatocyte growth factor effects in the modulation of in vitro survival and proliferation of germ cells during postnatal testis development [23].

## Conclusions

It has been demonstrated that LGF promotes germinal cell growth in male rat after ethane dimethanesulfonate (EDS)-induced testicular damage [6]. Here we have observed that intraperitoneal administration of LGF was able to restore spermatogenesis in mice sterilized with busulfan, suggesting that LGF can collaborate in the mobilization of testicular stem cells to restore spermatogenesis after germinal epithelium damage. As busulfan is an anticancer drug that affects fertility, the administration of LGF could be employed as a single treatment to induce the recovery of patients from busulfan treatment.

## Competing interests

The authors declare that they have no competing interests.

## Authors' contributions

MPC and EP performed most of the experimental animals and wrote the manuscripts; SPC, MIS, and MVTL performed the experimental analysis of the sperm and testis; JJDG and AGA supervised all the work and assisted in writing the manuscript. All authors read and approved the final manuscript.
